# Colloidal Stability and Cytotoxicity of Polydopamine-Conjugated Gold Nanorods against Prostate Cancer Cell Lines

**DOI:** 10.3390/molecules26051299

**Published:** 2021-02-28

**Authors:** Nouf N. Mahmoud, Hakam Aqabani, Suhair Hikmat, Rana Abu-Dahab

**Affiliations:** 1 Faculty of Pharmacy, Al-Zaytoonah University of Jordan, Amman 11733, Jordan; hakamaqabani@gmail.com (H.A.); suhair.jasim@zuj.edu.jo (S.H.); 2 School of Pharmacy, The University of Jordan, Amman 11942, Jordan

**Keywords:** gold nanorods, polydopamine, prostate cancer cells, anti-invasion, colloidal stability

## Abstract

Prostate cancer is one of the most common cancers in men. Cell invasion is an important step in the process of cancer metastasis. Herein, gold nanorods (GNRs) and polyethylene glycol (PEG)-coated GNRs were conjugated with polydopamine (PDA). The PDA-nanoconjugates demonstrated excellent colloidal stability upon lyophilization and dispersion in cell culture media with or without the addition of fetal bovine albumin (FBS), compared to unconjugated GNRs. PDA-nanoconjugates exhibited a considerable cytotoxicity against DU-145 and PC3 prostate cancer cell lines over a concentration range of 48 μg/mL–12 μg/mL, while they were biocompatible over a concentration range of 3.0 μg/mL–0.185 μg/mL. Furthermore, PDA-nanoconjugates demonstrated possible anti-invasion activity towards prostate cancer cell lines, particularly DU-145 cell line, by reducing cell migration and cell adhesion properties. The PDA-nanoconjugates could be considered a promising nano-platform toward cancer treatment by reducing the invasion activity; it could also be considered a drug delivery system for chemotherapeutic agents.

## 1. Introduction

The size and structure, in addition to the optical properties and surface plasmon resonance (SPR) of gold nanoparticles (GNPs), and the capability of their surface to be functionalized with a wide variety of ligands, make them strongly involved in drug delivery, diagnostics, therapy and biosensing [1]. Non-spherical gold nanoparticles, such as gold nanorods (GNRs), are considered a new generation of nanoplatforms to treat various types of cancer [2]. A lack of targeting and the severe toxicity caused by chemotherapeutic drugs remains as some of the most important limitations in cancer treatment advancement. Nanoparticle-based therapeutic systems have already been introduced into the market for the diagnosis and treatment of cancers. Many GNP-based nano-therapeutics have been extensively researched and tested as promising agents in cancer nanomedicine [3,4].

Prostate cancer is one of the most common cancers in men; typically, it is an adenocarcinoma as it originates primarily from the glandular part of the organ and shows glandular patterns on microscopic examination [5]. Metastasis of prostate cancers can complicate the disease and result in a poor prognosis. Tumor metastasis and invasion are complicated processes, where thousands of molecules and enzymes participate in the process during which interactions within tumor cells and their surroundings occur by direct cell-to-cell interactions and via secretion of several factors. During the metastasis cascade, multiple distinct steps are observed: loss of cell adhesion, increased motility and invasiveness, entry and survival during circulation, entry into the new tissue, and colonization at the distant site [6]. Invasion is an important step in the metastasis process, and consequently, drugs that can inhibit any part of the invasion cascade can play a role in the treatment of cancer. GNRs cause cancer cell destruction by various mechanisms, including photothermal destruction, induction of cell necrosis and apoptosis, metastatic cell suppression, and migration inhibition [7]. GNPs have been utilized to diagnose and treat prostate cancer in several studies [8,9,10,11]. However, few publications concerning the invasion inhibition properties of GNPs as a mono or combined therapy against prostate cancer cells.

Conjugation of GNPs with several ligands to enhance their anti-invasion activity has been investigated. For example, GNPs conjugated to a peptide can suppress breast cancer’s invasive activity [12]. Moreover, Ali et al. demonstrated that targeting GNPs to the cell nucleus region inhibited cancer cell migration and invasion [13].

Conjugation of GNPs with polydopamine (PDA) has been conducted previously to improve drug delivery and biocompatibility of GNPs and enhance their anti-cancer activity [14,15]. Previous studies demonstrated that when combining PDA-GNPs with anti-cancer agents such as paclitaxel [16] or doxorubicin [17], cancer treatment’s efficiency is drastically enhanced.

Dopamine (DA) can self-polymerize in an aqueous alkaline medium on the surface of various materials to form a surface coat of polydopamine. Borcherding et al. demonstrated that dopamine and its agonist suppressed cell viability, inhibited cell invasion, and induced apoptosis in breast cancer cell lines [18]. Nieto et al. demonstrated that the smaller PDA nanoparticle size is related to a more enhanced cytotoxicity against cancerous cells due to polydopamine’s affinity for iron ions [19]. In this work, the conjugation of polydopamine to GNRs and polyethylene glycol (PEG)-coated GNRs was optimized. The colloidal stability, cytotoxicity, and anti-invasion activity of the obtained PDA-nanoconjugates against two prostate cancer cell lines were investigated and reported.

## 2. Results and Discussion

### 2.1. Synthesis and Characterization of GNRs and Their Surface Functionalization with PEG-SH

**Figure 1:**
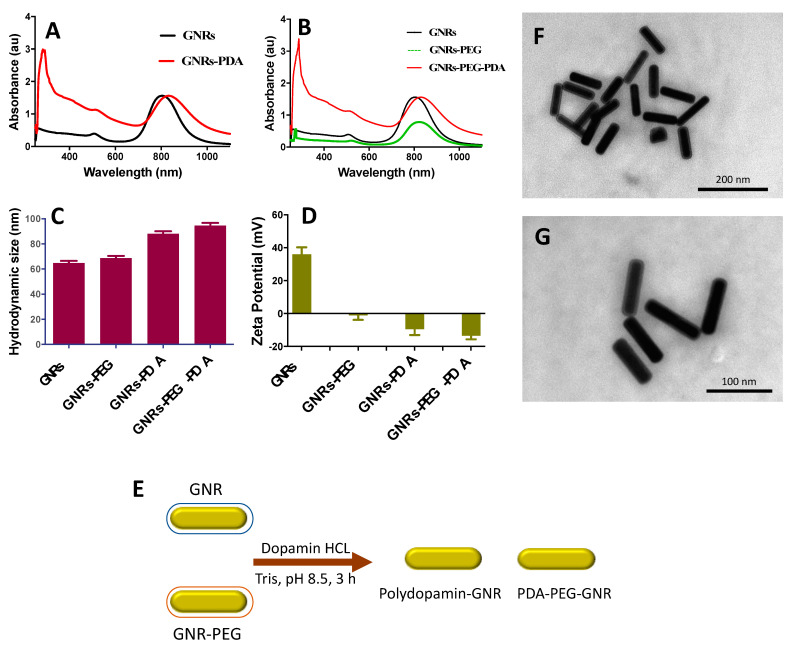
(**A**) UV-Vis absorption spectra of GNRs and GNRs-PDA. (**B**) UV-Vis absorption spectra of GNRs, GNRs-PEG and GNRs-PEG-PDA. (**C**) Hydrodynamic sizes of GNRs, GNRs-PEG, GNRs-PDA and GNRs-PEG-PDA. (**D**) Zeta potential values of GNRs, GNRs-PEG, GNRs-PDA and GNRs-PEG-PDA. (**E**) A Scheme demonstrated briefly the preparation of PDA-conjugated GNR. (**F**&**G**) TEM images of GNRs-PEG-PDA revealed an average length and width of 77.5 nm ± 5.5 nm and 19.4 nm ± 6.2 nm, respectively, and an average aspect ratio (AR) ~4. The thickness of the PDA layer around the nanoparticles is estimated to be ~6.1 nm ± 1.4 nm.

The UV-Vis absorption spectrum profile of GNRs revealed typical transverse and longitudinal peaks appeared at ~520 nm and ~820 nm, respectively, with no significant peak broadening or tailing, which indicates the excellent colloidal stability of the nanorods (Figure 1A). Functionalization of GNRs with PEG-SH resulted in a slight shift of the optical spectrum due to the surface coating (Figure 1B). The hydrodynamic size of the GNRs was ~65 nm, and it became ~70 nm upon PEGylation (Figure 1C). Furthermore, the surface functionalization efficiency was confirmed by the surface effective charge of the coated GNRs, where the zeta potential was reduced from +36 mV to +2 mV upon functionalization with PEG-SH (Figure 1D).

### 2.2. Surface Functionalization of GNRs with PDA

Both GNRs and GNRs-PEG were functionalized with PDA to compare the two conjugates in terms of their colloidal stability after conjugation and their cytotoxicity against prostate cancer cell lines (Figure 1E). Different parameters were optimized in order to obtain the most stable and effective PDA-conjugated GNRs.

The current conjugation methods of GNRs with PDA in the literature are highly inconsistent. There are many factors (such as concentration of dopamine and time of polymerization) affecting the properties of PDA-GNPs conjugates; particularly in their colloidal stability, uptake and cellular cytotoxicity [20]. One of the most common theories explaining the mechanism of PDA formation indicates that the formation of PDA coatings occurs by oxidative polymerization of dopamine, and the product; dopamine-quinone, undergoes a nucleophilic intramolecular cyclization reaction leading to the formation of 5,6-dihydroxyindole; and these products are the key building blocks for PDA [21]. The polymerization of DA on the surface of GNPs occurs in alkaline condition to form a PDA layer assembled on GNPs surface via coordination interaction between metal and catechol group [22].

In the current work, the polymerization of DA on the surface of GNRs was optimized using different parameters. For example, the Tris buffer concentration, DA, and GNRs greatly affected the conjugated nanoparticles’ stability and yield (Table 1). Furthermore, the reaction time significantly affected the stability of the conjugated nanoparticles and the degree of conjugation, which is confirmed by the red-shift of the longitudinal peaks of GNRs upon PDA functionalization. The temperature of the reaction affects the colloidal stability of the functionalized nanoparticles, whereas the room temperature is the best temperature to prevent nanoparticle aggregation (Table 1). Moreover, the preservation solution’s type and pH have a significant effect on the conjugated nanoparticles’ stability, where the best preservation solution for PDA-conjugated GNRs is phosphate buffer (pH 8.5).

UV-Vis absorption spectra of the synthesized GNRs-PDA displayed typical UV-Vis absorption peaks of GNRs. Functionalization of the GNRs and GNRs-PEG with PDA was resulted in a red-shift of the longitudinal peaks due to polymerization of DA on the surface of GNRs (Figure 1A,B). The significant increase in the hydrodynamic size of the functionalized GNRs confirms their successful surface coating with PDA; the hydrodynamic size of GNRs was increased from ~65 nm to ~88 nm after PDA conjugation, and the size of GNRs-PEG was increased from ~69 nm to ~95 nm upon conjugation with PDA (Figure 1C).

The surface effective charge of the coated GNRs confirms their surface functionalization efficiency; the zeta potential value was decreased from +33 mV to −11 mV upon surface coating of GNRs with PDA and was decreased from +2 to −12 mV upon conjugating the GNRs-PEG with PDA (Figure 1D). The PDA charge is due to the protonation/deprotonation of the phenolic and amino groups according to the pH of the solution; in our case, the PDA will be negatively charged at pH 8.5 [23].

The shape, size, dispersion, and surface functionalization of GNRs-PDA were verified by Transmission Electron Microscope (TEM) imaging which revealed a rod-shape of the nanoparticles with a thin layer around them that suggests the successful surface coating of the nanorods (Figure 1F,G).

**Figure 2:**
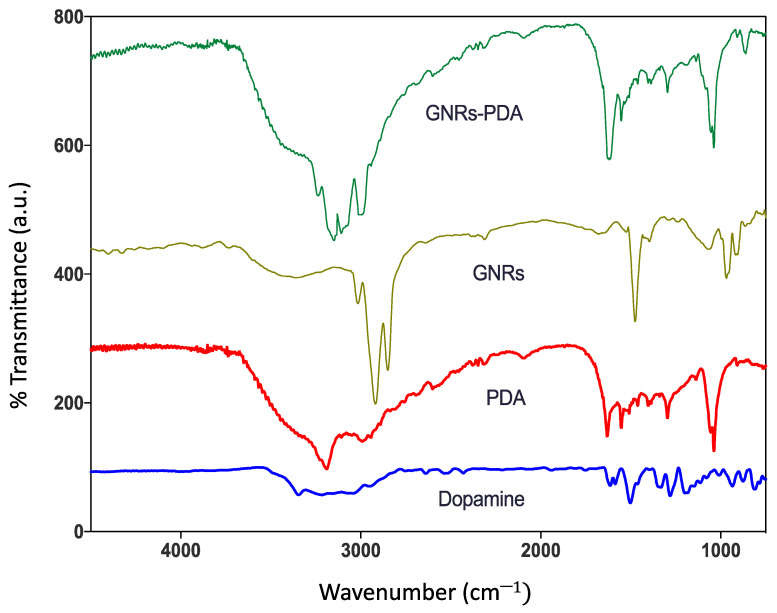
FTIR spectra of dopamine, polydopamine (PDA), GNRs and polydopamine-conjugated GNRs.

Fourier transform infrared (FTIR) analysis was performed to further confirm the successful conjugation of GNRs with PDA; the absorption band of PDA has the following distinct spectra; at ~3200 cm^−1^, which corresponds to the stretching vibrations of –OH and N–H groups in the PDA. Moreover, the sharp peaks at ~1629 cm^−1^ and ~1292 cm^−1^ are attributed to the C=O and C–O bonds, respectively. The peak at 1512 cm^−1^ is attributed for C=N and C=C. Upon conjugating GNRs with PDA, the FTIR spectrum of the conjugate, GNRs-PDA, demonstrated a similar spectrum to that of PDA (particularly the peaks related to the stretching vibrations –OH and N–H groups) and to that of the unconjugated GNR. However, the peaks were slightly broadened and underwent a low frequency due to conjugation with the surface of GNRs (Figure 2) [24].

### 2.3. The Colloidal Stability of GNRs, GNRs-PEG, GNRs-PDA and GNRs-PEG-PDA upon Lyophilization

Lyophilization usually affects the colloidal stability of nanoparticles and enhances their aggregation. In most cases, cryoprotectants should be added to enhance the colloidal stability of the nanoparticles and prevent their aggregation. The stability of the GNRs and GNRs-PEG and their corresponding PDA conjugates was compared before and after lyophilization in terms of their physical appearance, ease of reconstitution with phosphate buffer (pH = 8.5), and their optical spectra.

**Figure 3:**
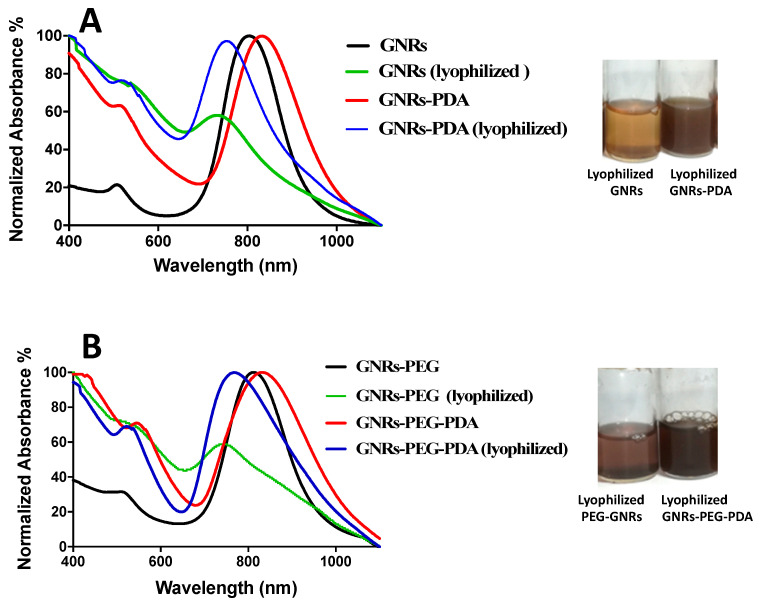
(**A**) UV-Vis absorption spectra of GNRs and GNRs-PDA before and after lyophilization. (**B**) UV-Vis absorption spectra of GNRs-PEG and GNRs-PEG-PDA before and after lyophilization. The photos indicate loss of the colloidal stable color and aggregation of the lyophilized GNRs and GNRs-PEG compared to lyophilized GNRs-PDA and GNRs-PEG-PDA, which have a superior colloidal stability.

Interestingly, both GNRs-PDA and GNRs-PEG-PDA exhibited superior stability upon lyophilization compared to other GNRs, confirmed by their colloidal color and optical spectra (Figure 3A,B). GNRs and GNRs-PEG exhibited broadening and tailing of the longitudinal peaks after lyophilization due to nanoparticle aggregation (Figure 3A,B). PDA-conjugated nanoparticles’ colloidal stability may be due to the shielding effect provided by PDA that keeps the nanoparticles away from each other and prevents their agglomeration.

### 2.4. The Colloidal Stability of GNRs upon Mixing with Cell Culture Medium with and without the Addition of Fetal Bovine Serum (FBS)

The components of cell culture media may retard or enhance nanoparticles’ colloidal stability through loss of the surface functionality or adsorption of protein/molecule on the nanoparticles’ surface [25]. Cell culture media are complex aqueous mixtures with different compositions according to several cell types’ different metabolic and nutritional needs [26]. Basically, all cell culture media are composed of amino acids, vitamins, inorganic salts and other components (such as glucose and sodium pyruvate) in different concentrations. Interaction of nanoparticles with the constituents of cell culture media could affect their cellular uptake and biological consequences.

The presence of proteins in cell culture media could significantly affect nanoparticles’ colloidal stability depending on the protein and nanoparticle concentrations, type of nanoparticles, and their physicochemical properties and ionic strength [25,27,28].

In this study, the effect of the Roswell Park Memorial Institute medium (RPMI) cell culture medium with and without the addition of FBS on the stability of nanoparticles was examined by measuring the optical spectra at different time points of incubation observing the colloidal color of the nanoparticles and their dispersibility. The GNRs plasmon resonance composed of two bands of absorption, the transverse (at ~530 nm), and longitudinal plasmons (600–1100 nm); the longitudinal plasmon is more sensitive to nanoparticles aggregation than the transverse one, and it is considered as a robust indicator of nanoparticles’ agglomeration [29].

**Figure 4:**
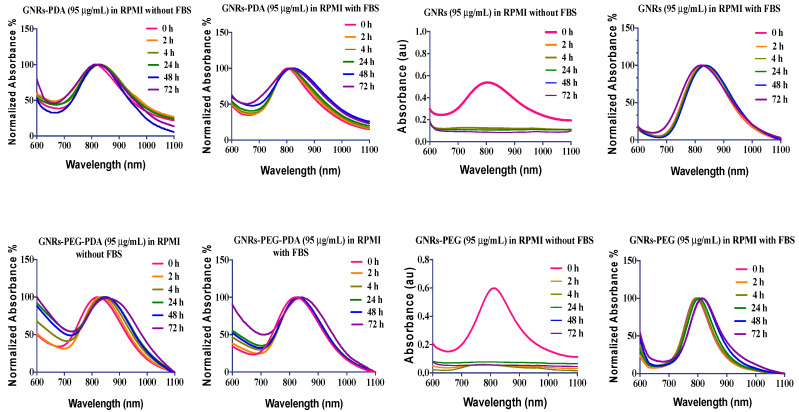
The longitudinal optical spectra of GNRs, GNRs-PEG, GNRs-PDA, GNRs-PEG-PDA dispersed in RPMI cell culture medium with and without addition of FBS after different incubation times.

The results presented in Figure 4 demonstrated that GNRs and GNRs-PEG showed typical and stable longitudinal peaks upon mixing with FBS-containing cell culture media over 72 h of incubation; mixing GNRs GNRs-PEG with FBS-free medium lead to complete aggregation of the nanoparticles. Interestingly, conjugating GNRs with PDA (GNRs-PDA and GNRs-PEG-PDA) greatly enhanced the conjugates’ colloidal stability upon mixing with cell culture medium with or without the addition of FBS. We demonstrated the crucial role of FBS in cell culture media in preserving the nanoparticles’ colloidal stability of several surface chemistries [30]. PDA-coated gold nanospheres were stable in protein-containing cell culture media; however, their stability was collapsed upon dispersion in protein-free media [20].

FBS main contains bovine serum albumin (BSA) in addition to other different proteins. Electrostatic interactions, hydrophobic interactions, or specific chemical interactions are responsible for forming the protein corona [31]. The type of interaction between proteins and nanoparticles depends on the pH and ionic strength of the medium. In our work, the medium’s pH is above the isoelectric point (IEP) of BSA; thus, electrostatic interactions are expected to occur between the protein and the nanoparticles, particularly the positively charged ones. The formation of a protein corona on the surface of neutral and negatively charged GNR is expected too, due to other components and proteins in FBS [31,32].

The zeta potential of the nanoparticles dispersed in FBS-containing cell culture media ranged from +1.4 to −6 and for those dispersed in FBS-free media, the surface charge ranged from +0.4 to −4. For mixtures without FBS, the reduction in zeta potential values was due to the medium’s high ionic strength that induced aggregation by neutralizing the surface charges. However, for mixtures with FBS, the nanoparticles’ surface charges were neutral too; this is most likely due to the steric repulsion provided by adsorption of FBS proteins on the surface of nanoparticles and the formation of a protein corona [33]. In the extended DLVO theory, factors other than electrostatic repulsion like steric and hydration repulsions are introduced which affect the colloidal stability [34]. The nanoparticles’ size was drastically increased for unconjugated nanoparticles dispersed in FBS-free cell culture medium.

The colloidal stability of various nanoparticles is dependent on the nanoparticles’ composition and the concentration of FBS added [25,32,35,36,37]. Basuki et al. showed that poly(oligoethylene glycol acrylate)-functionalized iron oxide nanoparticles were not stable in FBS-containing media [38]. On the other hand, lysozyme-loaded lipid-polymer hybrids exhibited no significant change in nanoparticle size and charge when incubated with only medium or 10% FBS-containing medium [39]. The stabilization effect of FBS in our study is most likely related to the formation of a protein corona by either electrostatic or hydrophobic attraction depending on the nanoparticles’ charge and pH of the medium. Interestingly, nanoparticles conjugated with PDA demonstrated excellent colloidal stability in the cell culture medium with or without the addition of FBS, which is most likely due to the steric repulsion provided by PDA [40].

### 2.5. Antiproliferative Activity of GNRs against PC3 and DU-145 Prostate Cancer Lines

Numerous abnormal biological processes occur at the cellular and sub-cellular levels during prostate cancer initiation, progression, and relocation. These involve cell death and survival, cell invasion and metastasis, and dysregulation of many interrelated signaling pathways.

PC3 and DU-145 cell lines are prostate cancer cell lines established from metastatic deposits (central nervous system and bone/lumbar spine, respectively). Both of them lack the androgen receptor and are androgen-independent. PC3 cells are more tumorigenic and have a higher metastatic potential than DU-145 [41]. PC3 and DU-145 cell lines are considered the golden standard for testing newer drugs/delivery systems for potential anticancer activity against prostate cancer.

**Figure 5:**
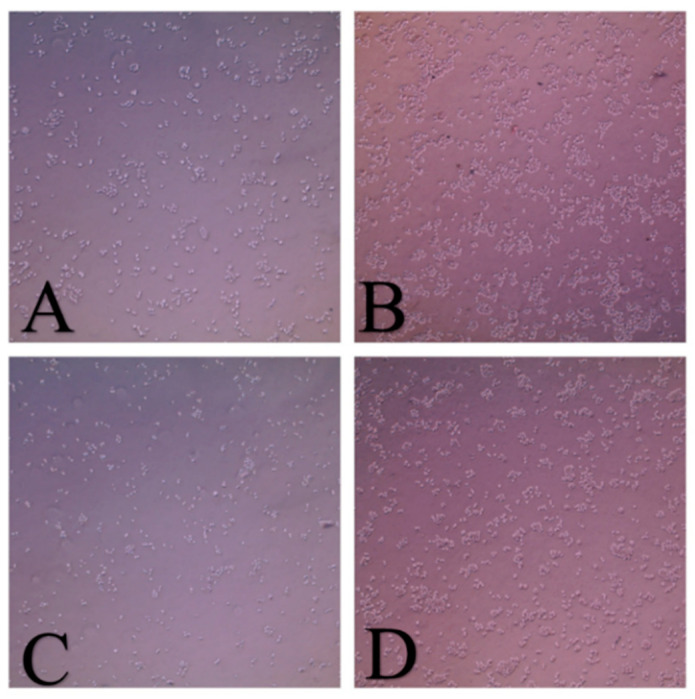
Cell culture in RPMI medium with and without addition of FBS for 72 h; (**A**) DU-145 in FBS-free medium; (**B**) DU-145 in RPMI with 10% FBS; (**C**) PC3 in FBS-free medium; and (**D**) PC3 in FBS-containing medium.

According to the previous colloidal stability studies, the antiproliferative activity of GNRs and GNRs-PEG and their corresponding PDA-conjugates was examined in FBS-containing cell culture media. Figure 5 showed good cellular growth in FBS-containing media (Figure 5B,D) compared to those grown in FBS-free medium (Figure 5A,C).

**Figure 6:**
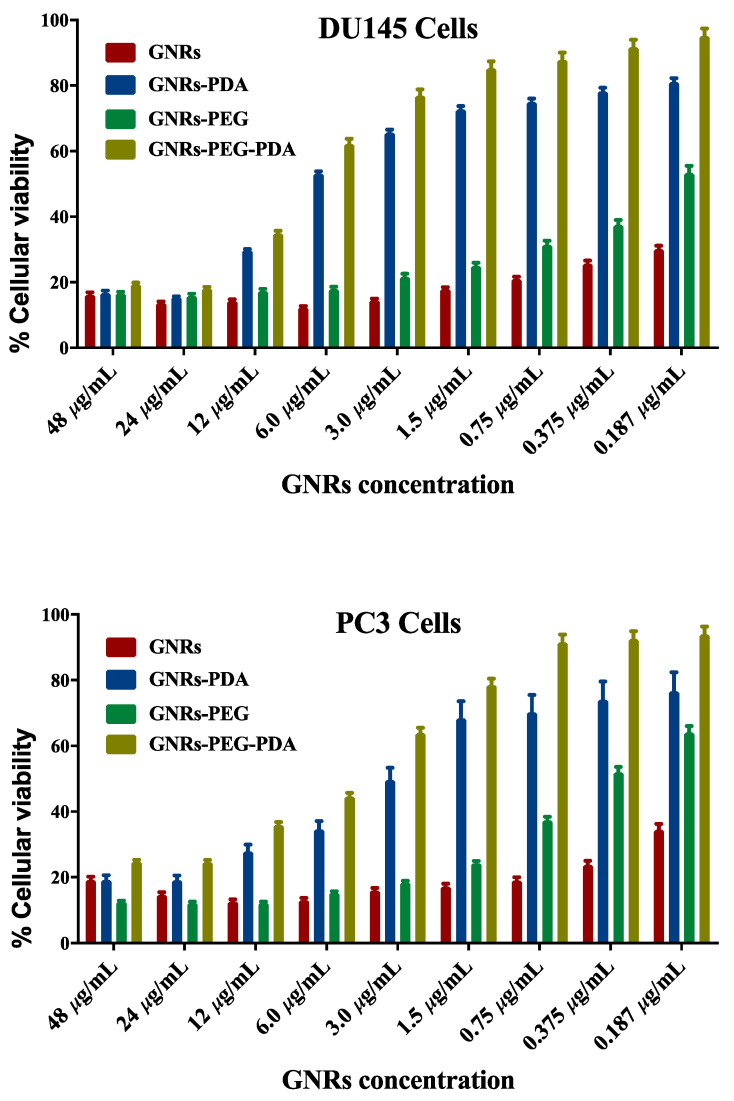
Cellular viability of PC3 and DU-145 prostate cancer cell lines upon incubation with GNRs, GNRs-PEG, GNRs-PDA and GNRs-PEG-PDA for 72 h.

The cellular viability of both cell lines treated with GNRs and GNRs-PEG and their corresponding PDA-conjugates was investigated using MTT assays. As demonstrated in Figure 6, unconjugated GNRs exhibited substantial cytotoxicity (18–25% cellular viability) over a wide range of concentrations against both cell lines; however, PEGylation of GNRs has slightly reduced the cytotoxicity of the nanoparticles against DU-145 (20–50% cellular viability) and PC3 cell lines (20–60% cellular viability). Conjugating GNRs or GNRs-PEG with PDA reduced their cytotoxicity against both cell lines (20–90% cellular viability), over the low concentration range (3.0 μg/mL–0.185 μg/mL) after 72 h of incubation. However, PDA-conjugates preserved their cytotoxicity against both cell lines over the concentration range of 48 μg/mL to 12 μg/mL. PDA conjugated with PEGylated GNRs (GNRs-PEG-PDA) demonstrated slightly higher cellular viability than PDA conjugated to uncoated GNRs (GNRs-PDA) over the low concentration range. We propose that conjugating the nanoparticles with PDA may reduce their cellular uptake and consequently their direct cytotoxicity at low concentrations. PDA-coated nanoparticles’ biocompatibility and their utilization in various biomedical applications such as drug delivery, imaging, and photothermal therapy were demonstrated in several studies [42,43]. However, the crucial role of nanoparticles’ size and type of cell line exposed to treatment was reported in determining the cytotoxicity of conjugated nanoparticles [44]. Further, it was reported that the time of polymerization of DA to PDA onto the surface on GNPs greatly affected the cellular uptake extent of the PDA conjugates; those prepared by short polymerization time (1–6 h) were highly internalized into the cells compared to those prepared by using extended polymerization [20]. In our study, short polymerization time was used to prepare the PDA-conjugates.

On the other hand, it is well-known that the charge of nanoparticles can influence their cellular internalization, consequently their toxicity towards cells; negatively charged nanoparticles in most cases induce low cytotoxicity due to their reduced cellular uptake compared to positively and neutral-charged nanoparticles [45]. This could explain our PDA-conjugates’ low cytotoxicity, particularly at low concentrations compared to their unconjugated GNRs counterparts.

### 2.6. In Vitro Cell Migration Assay of Prostate Cancer upon Treatment with GNRs-PDA and GNRs-PEG-PDA

The anti-invasion activities of GNRs conjugated with PDA towards prostate cancer cells were investigated using two different approaches. First, we investigated the effect of GNRs-PDA and GNRs-PEG-PDA (at concentrations that achieved cellular viability of around 80%) on preventing cell migration using the scratch assay.

The results showed that untreated PC3 and DU-145 cell lines demonstrated complete cell migration and formation of cell monolayers after 48 h of incubation. However, the scratch in cells treated with quercetin as a positive control was kept open after 48 h of treatment due to its well-known ability of migration inhibition [46].

**Figure 7:**
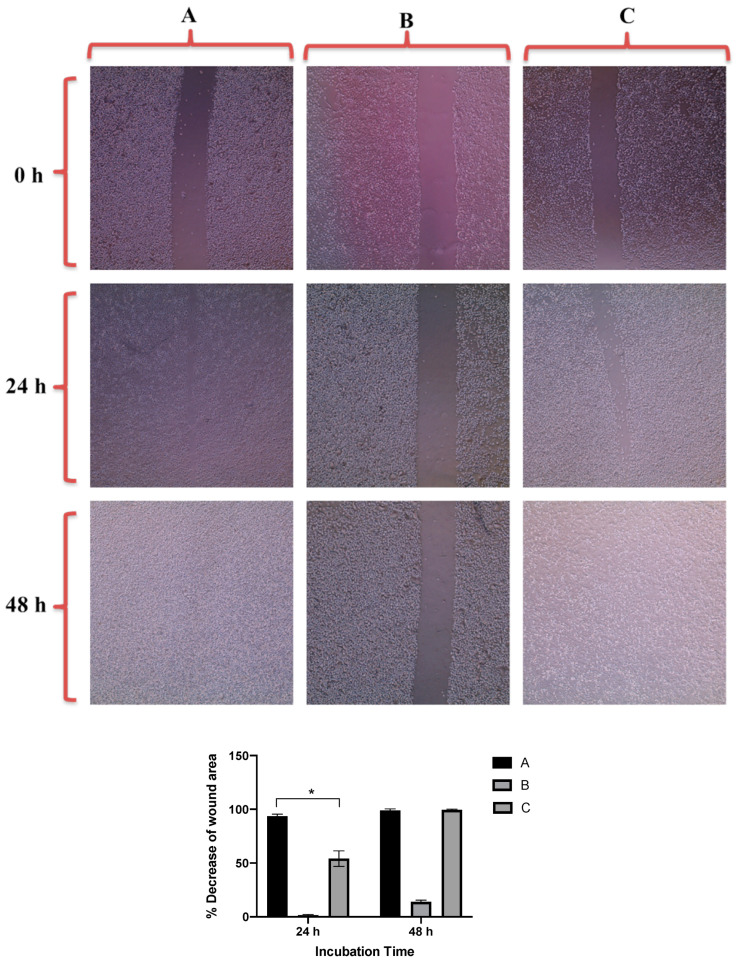
Up: In vitro cell migration of PC3 cell line (control untreated cells) (**A**), upon treatment with positive control (quercetin) (**B**), and upon treatment with GNRs-PDA (**C**). Magnification power: 10×. Down: The percentage decrease in wound area was estimated using ImageJ. Data are represented as mean ± standard deviation (SD), *n* = 3. *t*-test was employed for the difference assessment; * *p* < 0.05.

**Figure 8:**
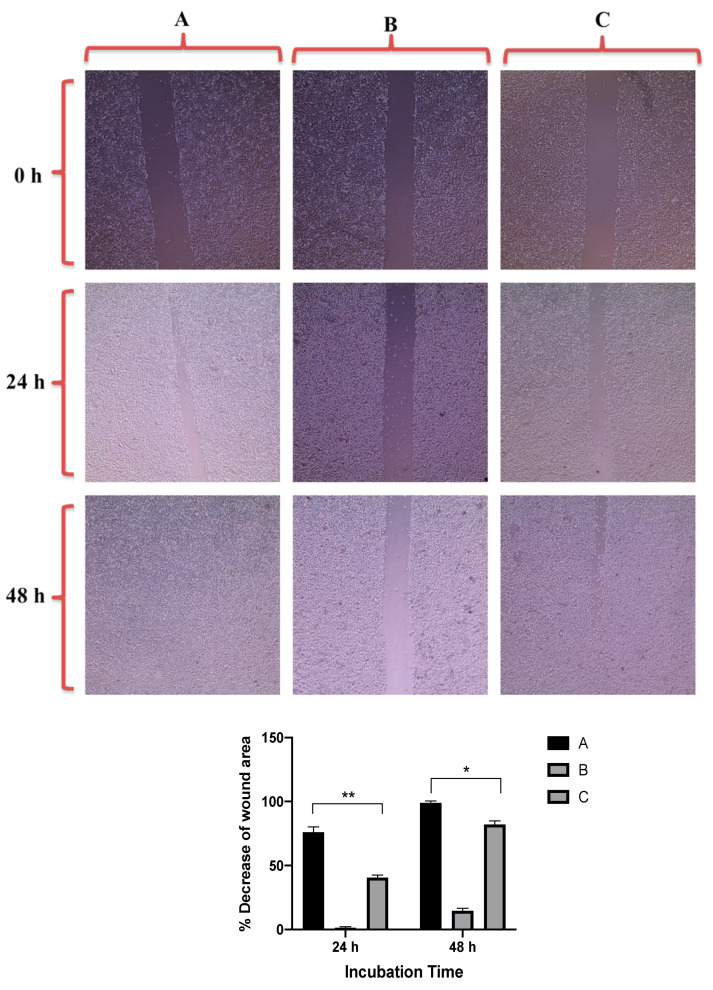
Up: In vitro cell migration of DU-145 cell line (control untreated cells) (**A**), upon treatment with positive control (quercetin) (**B**), and upon treatment with GNRs-PDA (**C**). Magnification power: 10×. Down: The percentage decrease in wound area was estimated using ImageJ. Data are represented as mean ± standard deviation (SD), *n* = 3. *t*-test was employed for the difference assessment; * *p* < 0.05, ** *p* < 0.01.

**Figure 9:**
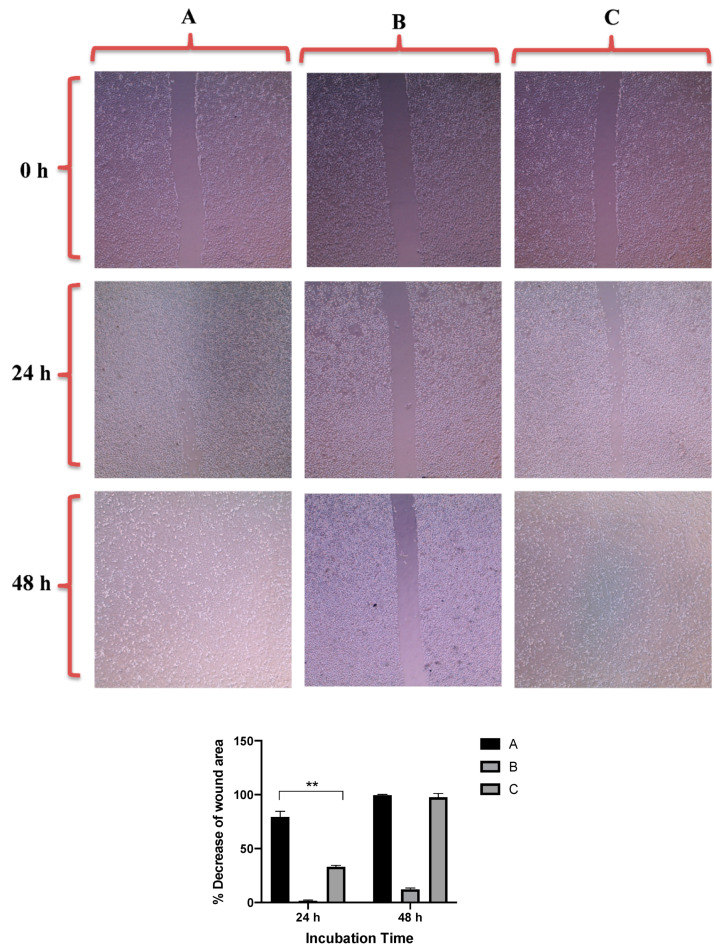
Up: In vitro cell migration of PC3 cell line (control untreated cells) (**A**), upon treatment with positive control (quercetin) (**B**), and upon treatment with GNRs-PEG-PDA (**C**). Magnification power: 10×. Down: The percentage decrease in wound area was estimated using ImageJ. Data are represented as mean ± standard deviation (SD), *n* = 3. *t*-test was employed for the difference assessment; ** *p* < 0.01.

**Figure 10:**
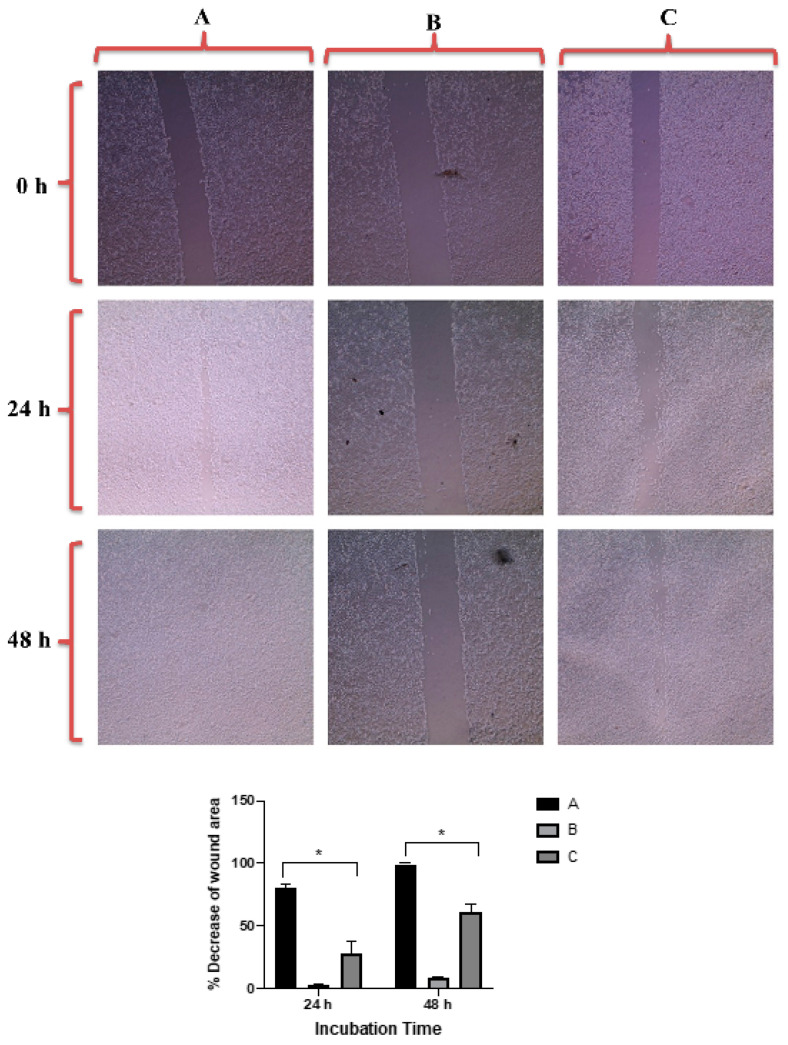
Up: In vitro cell migration of DU-145 cell line (control untreated cells) (**A**), upon treatment with positive control (quercetin) (**B**), and upon treatment with GNRs-PEG-PDA (**C**). Magnification power: 10×. Down: The percentage decrease in wound area was estimated using ImageJ. Data are represented as mean ± standard deviation (SD), *n* = 3. *t*-test was employed for the difference assessment; * *p* < 0.05.

Figure 7 revealed that GNRs-PDA retarded the cell migration potential of PC3 cell line after 24 h of incubation compared to the untreated cells (54% vs. 93%, average reduction of wound area). However, the inhibition of migration was not significant after 48 h compared to untreated cells. On the other hand, GNRs-PDA reduced the cell migration potential of DU-145 cell line after 24 h (40% vs. 76%, average reduction of wound area), and 48 h (80% vs. 99%, average reduction of wound area) of incubation compared to the control untreated cells (Figure 8). Similarly, GNRs-PEG-PDA significantly reduced the cell migration potential of PC3 cells after 24 h of incubation compared to untreated cells (33% vs. 80%, average reduction of wound area), however, both untreated and treated PC3 cells exhibited similar cell migration and formation of monolayers after 48 h of incubation (Figure 9). On the other hand, DU-145 cells treated with GNRs-PEG-PDA demonstrated reduced cell migration efficiency compared to untreated cells after 24 h (28% vs. 80%, average reduction of wound area), and 48 h (60% vs. 99%, average reduction of wound area) of incubation (Figure 10).

GNPs exhibit anticancer activity against different cancer cells by several mechanisms [47]. GNPs combined with radiation revealed inhibition of both PC3 and DU-145 cells [48]. A recent study indicated that PDA conjugation with gold nanostars inhibited proliferation, migration, and tube formation of human umbilical vein endothelial cells and enhanced the photothermal effect and drug delivery properties of the carrier [49].

**Figure 11:**
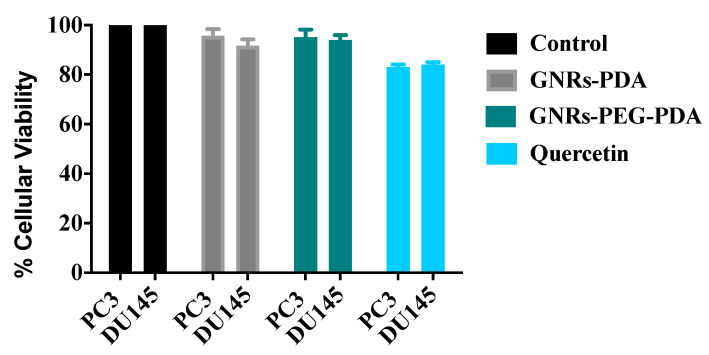
Cellular viability of GNRs containing PDA towards PC3 and DU-145 prostate cancer cells compared to positive and negative controls after scratch assay.

The cellular viability of both cell lines was estimated after scratch assay to ensure that the nanoparticles’ observed anti-migration activity was not due to their cytotoxicity towards cells. The results presented in Figure 11 indicated that both cell lines demonstrated high cellular viability upon treatment with positive control and PDA conjugates.

### 2.7. Evaluation of the In Vitro Adhesion Assay upon Treatment with GNRs-PDA and GNRs-PEG-PDA

Another approach to explore the inhibition of invasion and metastasis potential of the nanoconjugates is to investigate their ability to inhibit the epithelial-mesenchymal transition (EMT). EMT is a critical process involving the initiation, growth, invasion, and metastasis of cancer. EMT depends on various cellular functions, including the reduction in expression of cell adhesion molecules [50]. In metastatic cancers, cell adhesion undergoes rapid regulatory changes that allow the cancer cells to disengage from the extracellular matrix (ECM), migrate and then reengage with the ECM at its secondary metastatic site. Inhibition of adhesion could be another area to inhibit cancer cell metastasis.

Anti-adhesion activities of GNRs conjugated with PDA have been investigated using adhesion assays on both cell lines, at the same concentrations used in scratch assays, to study their possible effect on cell adhesion compared to the positive control (quercetin). The results shown as a percent adhesion inhibition using 3-(4,5-Dimethylthiazol-2-yl)-2,5-diphenyltetrazolium bromide (MTT) assay as a quantitative evaluation of cell adhesion.

**Figure 12:**
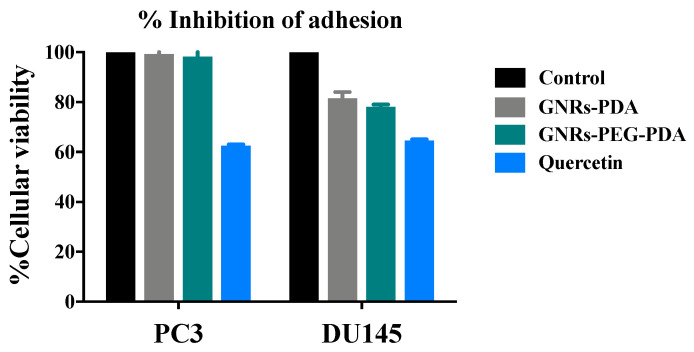
Percent inhibition of adhesion of GNRs-PDA and GNRs-PEG-PDA compared to untreated cells (control) and positive control (Quercetin).

The percent inhibition of adhesion of PC3 and DU-145 cell lines upon exposure to GNRs-PDA, GNRs-PEG-PDA and quercetin are demonstrated in Figure 12. The results show that GNRs conjugated with PDA demonstrated moderate anti-adhesion potential against DU-145 cell line (18–22%) in comparison to control; however, no significant inhibition of adhesion was observed for the PC3 cell line.

Both anti-adhesion and scratch assay results reveal that GNRs conjugated with PDA may have anti-invasion activity, particularly towards the DU-145 cell line. Although PC3 and DU-145 are both androgen-independent prostate cancer, some studies suggest that the difference of migration potential response between the two cell lines could be related to TRPM8 receptors [51,52,53], whereas DU-145 cells are the most sensitive cell type to migration inhibition by TRPM8 blocking [54]. It was reported that activation of the DAR receptor by dopamine may lead to modulation in the Ca^2^^+^ level, which acts as a channel blocker of TRPM8 [55].

Moreover, the difference in response between DU-145 and PC3 cell lines may be related to many other reasons; DU-145 cell line has higher level of basal GSH content and GSH/GSSG ratio than that of PC3 cell line [56], and both cell lines showed differences of receptor activation such as MMP2 and MMP9 [57].

## 3. Materials and Methods

### 3.1. Materials

The following materials were obtained from Sigma Aldrich Chemicals (St. Louis, MO, USA): chloroauric acid 99.9% (HAuCl_4_·3H_2_O); silver nitrate 99% (AgNO_3_); methoxy PEG-thiol (m-PEG-SH, MW ~2000 g/m); sodium borohydride 99% (NaBH_4_); sodium oleate (NaOL); L-ascorbic acid (99.9%); potassium bromide for IR (KBr); fetal bovine serum (FBS); Tris (2-amino-2-(hydroxymethyl)-1,3-propanediol) base; 3-hydroxytyramine hydrochloride (dopamine·HCl, 99.9%). Prostate cancer cell lines—ATCC^®^ CRL-1435TM (PC3) and ATCC^®^ HTB-81TM (DU 145)—were obtained from ATCC (Manassas, VA, USA). 3-(4,5-Dimethylthiazol-2-yl)-2,5-diphenyltetrazolium bromide (MTT) was obtained from Promega (Madison, WI, USA). The following materials were obtained from Euro-Clone^TM^ (Pero, MI, Italy): Dimethyl sulfoxide (DMSO) cell culture grade; penicillin-streptomycin solution, 100×; Roswell Park Memorial Institute medium (RPMI-1640); Trypan blue 0.5%; Trypsin-EDTA 0.2% in PBS. Gentamycin 10 mg/mL was obtained from Capricorn Scientific (Ebsdorfergrund, Germany). Phosphate buffered saline (PBS) was obtained from Eurobio (Les Ulis, France).

The following instruments and equipment were used in this work: Electronic balance, Shimadzu (Kyoto, Japan); EVOS™ XL Core Configured Microscope AMEX1200, Thermo Fischer Scientific (Waltham, MA, USA); Incubator Avantgarde (Munich, Germany); Fourier transformed infrared (FT-IR) spectroscopy, Shimadzu (Kyoto, Japan); Hermle Z230A centrifuge, Wehingen, Germany); Malvern Zeta sizer ZS 90 particle size/zeta potential analyzer (Particle Sizing Systems, Santa Barbara, CA, USA). Multi-mode microplate reader BioTek, (Winooski, VT, USA); pH meter, Hanna Instruments (Woonsocket, RI, USA); Free Zone Plate shaker, Boekel Scientific 130,000 (Vernon Hills, IL, USA); UV-1800, UV-VIS spectrophotometer Shimadzu (Kyoto, Japan); Morgani 268 TEM, FEI (Eindhoven, The Netherlands).

### 3.2. Methods

#### 3.2.1. Synthesis of GNRs

GNRs were synthesized using a mixture of CTAB and sodium oleate following a previous protocol [58,59].

#### 3.2.2. PEGylating of GNRs (GNRs-PEG)

Thiolated PEG (PEG-SH) was used to functionalize the surface of GNRs by mixing each 1.0 mL of twice-cleaned GNRs with 0.1 mL of PEG solution (10 mg/mL) for 24 h. The resulted coated GNRs were centrifuged at 10,000 rpm for 10 min to remove the excess of PEG-SH polymer.

#### 3.2.3. Surface Functionalization of GNRs with PDA

The method of surface functionalization of GNRs with PDA was optimized in terms of the following parameters; concentration of Tris buffer (0.1 M and 0.01 M), concentration of GNRs (135 μg/mL and 90 μg/mL), concentration of DA (0.5–2 mg/mL), time of reaction (1, 3, 6 and 24 h), temperature of reaction (25 °C, 50 °C and 90 °C) and type and pH of the preservation solution (phosphate buffer or mill-Q water) (Table 1).

The optimized and successful surface functionalization of GNRs with PDA was performed as follows: A stock solution of Tris buffer (0.1 M, pH 8.5) was prepared. A volume of 6 mL of concentrated GNRs or GNRs-PEG was added to 44 mL of Tris buffer solution (0.01 M) to produce two solutions of GNRs (135 μg/mL). The GNRs solutions were kept on a magnetic stirrer for 5 min. Into each solution, 50 mg of DA dispersed in 0.5 mL ultrapure water was injected rapidly. The solutions were left on magnetic stirrer for 3 h at 25 °C and sonicated for 10 min every 30 min of reaction time. The reaction was performed in a dark place and free access to air. After 3 h, the solutions were centrifuged twice for 15 min at 12,000 rpm to collect the pellets. The produced pellets were re-suspended immediately in phosphate buffer (pH 8.5), to prevent nanoparticle aggregation.

#### 3.2.4. Characterization of the GNPs

The synthesized GNRs were characterized by measuring the zeta potential and hydrodynamic size of the nanoparticles. A UV-vis spectrometer was used to measure the UV-vis absorption spectra of GNRs over the range of 400–1100 nm wavelength. TEM imaging was used to confirm the size and structure of the nanoparticles by drying 8 μL GNPs on a Formvar coated TEM copper grid.

ImageJ 1.51 was used to estimate the mean length and width of synthesized GNRs. FTIR spectroscopy was used to confirm the surface functionalization of GNRs with the PDA, and potassium bromide disks were used to prepare samples (after lyophilization) for FTIR measurements.

The concentration of GNRs was measured by a validated method of inductively coupled plasma-optical emission spectroscopy (ICP-OES) at a wavelength of 242.795 nm and using a calibration curve of a gold standard for ICP, 1000 ppm (0.2−10.0 ppm, correlation coefficient (r^2^) = 0.9999).

#### 3.2.5. Lyophilization (Freeze-Drying) of GNRs

In the freeze-drying process, 15 mL glass vials were filled with 8 mL of the synthesized GNRs solutions, and the samples were frozen at −80 °C for 1 h, then placed in the freeze-dryer. The samples in the drying chamber were cooled below −50 °C, with application of vacuum (around 5 × 10^−3^ bar) overnight. The frozen-dried products were evaluated visually for their appearance, and then reconstituted with phosphate buffer. The reconstituted GNRs solutions were then analyzed by UV-vis absorption spectroscopy. The experiment was performed in triplicate.

#### 3.2.6. Colloidal Stability of GNRs in Cell Culture Media

The prepared GNRs (95 μg/mL) were mixed with RPMI cell culture medium with and without addition of 10% FBS. The GNRs-media mixtures were incubated at 37 °C for 72 h. Samples from the mixtures were taken at different time points; 2, 4, 24, 48 and 72 h, and the optical absorption spectra over 600–1100 nm, zeta potential, and hydrodynamic size were measured to evaluate the colloidal stability and dispersibility of the nanoparticles in the cell culture media. The experiment was performed in triplicate.

#### 3.2.7. Anti-Proliferative Activity of GNRs against Prostate Cancer Cell Lines

##### Cell Culture

DU-145 and PC3 prostate cell lines were cultured in RPMI medium and supplemented with L-glutamine (1.0%, 2.0 mM), FBS (10.0% *v*/*v*), penicillin (100 U/mL), streptomycin (100 μg/mL), and gentamycin (1.0 mL), at 5% CO_2_ and 99% relative humidity at 37 °C. The cells were stained after confluency with trypan blue dye (0.04%) and counted by a hemocytometer.

##### Anti-Proliferative Assay

A volume of 100 μL of the cell suspension of 5 × 10^3^ cells/well of both prostate cancer cell lines was seeded in 96-well plates and incubated for 24 h before the addition of GNRs suspension. A volume of 100 μL of each GNRs suspension (GNRs, PEG-GNRs, GNRs-PDA and GNRs-PEG-PDA) over a range of concentration (48 μg/mL–0.184 μg/mL) was added to the wells with the addition of 10% FBS.

For viability assay, the medium was removed carefully from the cells and 100 µL of fresh media and 10 µL of MTT (5 mg/mL) were added into each well. The plates were incubated for 4 h in 5% CO_2_ incubator and after incubation, the medium from the wells was removed carefully and 100 µL of DMSO was added to each well and mixed well by shaking for 20 min. The viable cells were measured by the development of purple color due to the formation of formazan crystals. The absorbance was recorded at 570 nm by multi-mode microplate reader, and the cellular viability percentage of the treated cells was calculated relative to the cellular viability of the control untreated cells. The experiment was performed in triplicate.

##### In Vitro Cell Migration Assay of Prostate Cancer Cell Lines upon Treatment with GNRs

The cell-cell interaction and their migration potential were investigated using cell migration assay. PC3 and DU-145 prostate cells were seeded into 6-well culture plates with 250 × 10^4^ cells per well. Then, the cells were incubated at 37 °C and 5% CO_2_ for 24 h to achieve more than 95% confluency and formation of an attached cells monolayer. Then, the monolayer was carefully scratched using a sterile plastic 200-µL pipette tip to draw a linear wound in the cell monolayer of each well. The wounded monolayers were washed twice with PBS to remove any cell debris. The wounded cells were treated with GNRs-PDA (0.361 μg/mL) or GNRs-PEG-PDA (0.741 μg/mL). Untreated cells were used as a negative control. Quercetin was used as a positive control (30 µM). The cultures were incubated at 37 °C, 5% CO_2_, and the wounds were carefully observed using phase contrast microscope at zero time, and after 24 h and 48 h. The percentage decrease in the wound area was estimated by using ImageJ version 1.51. The experiment was performed in triplicate.

##### In Vitro Adhesion Assay of Prostate Cancer Cell Lines upon Treatment with GNRs

Before adhesion test was conducted, viability test of both cell lines (PC3 and DU-145) was done to select a non-toxic concentration of the treatments. Cell viability reduction up to 10% was considered acceptable. Cells were grown in RPMI medium with addition of 10% FBS to achieve 80% confluence prior to beginning of the test. A 96-well plate was layered with 50 μL of fibronectin and incubated at 37 °C overnight, the next day, excess fibronectin was drained, and the plate was blocked with 50 μL of 0.2% bovine serum albumin (BSA). Cells were collected with trypsin/EDTA and re-suspended in RPMI medium at approximately 125,000 cells/mL, and 100 μL of cell suspension were applied to each well pre-coated with fibronectin. The experiment was repeated in triplicates for GNRs-PDA (0.361 μg/mL), GNRs-PEG-PDA (0.741 μg/mL), and the positive control, quercetin (30 µM). The plate was then incubated for 30 min at 37 °C. After the incubation period, the wells were gently aspirated and washed three times with PBS, then; assay was performed to quantify the viable cells [60]. The experiment was performed in triplicate.

## 4. Conclusions

In this study, GNRs were successfully conjugated with PDA. The GNRs-PDA and GNRs-PEG-PDA conjugates demonstrated excellent colloidal stability upon lyophilization and mixing with the cell culture medium. The cellular viability study demonstrated that PDA-conjugated GNRs exhibited considerable cytotoxicity over the concentration range of 48 μg/mL to 12 μg/mL and low cytotoxicity over the concentration range of 3.0 μg/mL to 0.185 μg/mL against both PC3 and DU-145 prostate cancer cell lines. The PDA conjugates reduced the cellular invasion potential of prostate cancer cell lines, particularly DU-145 cells, by retarding their cell migration and adhesion potentials. In addition to their possible contribution to prostate cancer therapy, PDA-conjugated GNRs could be considered stable drug delivery platforms for chemotherapeutic agents and treat cancers by photothermal properties.

## Figures and Tables

**Table 1:** Parameters used for production of PDA-conjugated GNRs.

Parameter			Other Conditions	Results
1.	Tris buffer concentration	0.1 M	135 μg/mL GNRs, 1 mg/mL DA, 3 h reaction, 25 °C	Nanoparticles aggregation
		0.01 M	135 μg/mL GNRs, 1 mg/mL DA, 3 h reaction, 25 °C	Stable conjugated GNRs
2.	GNRs concentration	90 μg/mL	0.01 M Tris, 1 mg/mL DA, 3 h reaction, 25 °C	Low yield of conjugated GNRs
		135 μg/mL	0.01 M Tris, 1 mg/mL DA, 3 h reaction, 25 °C	High yield of conjugated GNRs
3.	Dopamine concentration	0.5 mg/mL	0.01 M Tris, 135 μg/mL GNRs, 3 h reaction, 25 °C.	No PDA conjugation to GNRs
		1 mg/mL	0.01 M Tris, 135 μg/mL GNRs, 3 h reaction, 25 °C.	Successful PDA conjugation to GNRs
		2 mg/mL	0.01 M Tris, 135 μg/mL GNRs, 3 h reaction, 25 °C.	Nanoparticles aggregation
4.	Time of reaction	1 h	0.01 M Tris, 135 μg/mL GNRs, 1 mg/mL DA, 25 °C.	No PDA conjugation to GNRs
		3 h	0.01 M Tris, 135 μg/mL GNRs, 1 mg/mL DA, 25 °C.	Successful PDA conjugation to GNRs
		6 h	0.01 M Tris, 135 μg/mL GNRs, 1 mg/mL DA, 25 °C.	Successful PDA conjugation to GNRs
		24 h	0.01 M Tris, 135 μg/mL GNRs, 1 mg/mL DA, 25 °C.	Nanoparticles aggregation (slight)
5.	Temperature of reaction	25 °C	0.01 M Tris, 135 μg/mL GNRs, 1 mg/mL DA, 3 h reaction.	Successful PDA conjugation to GNRs
		50 °C	0.01 M Tris, 135 μg/mL GNRs, 1 mg/mL DA, 3 h reaction.	Nanoparticles aggregation
		90 °C	0.01 M Tris, 135 μg/mL GNRs, 1 mg/mL DA, 3 h reaction.	Nanoparticles aggregation
6.	Preservation solution	Phosphate buffer (pH 8.5)	0.01 M Tris, 135 μg/mL GNRs, 1 mg/mL DA, 3 h reaction, 25 °C.	Successful PDA conjugation to GNRs
		Ultrapure water	0.01 M Tris, 135 μg/mL GNRs, 1 mg/mL DA, 3 h reaction, 25 °C.	Nanoparticles aggregation

## Data Availability

Data are available on request from the corresponding authors.

## Sample Availability

Samples of the compounds are not available from the authors.
